# Supplementary material for: Therapeutic targeting of oligodendrocytes in an agent-based model of multiple sclerosis
Source: PLoS Comput Biol. 2026 Jan 20;22(1):e1013273. doi: 10.1371/journal.pcbi.1013273 (PMC12844537; doi:10.1371/journal.pcbi.1013273)
Supplement: S1 File — (PDF) [file pcbi.1013273.s001.pdf]

S1 Supporting Information

Full parameter table

We have extensively searched the literature to sensibly parameterise the model. All parameters are given in Table A. Note that some parameters are estimated and/or subjected to sensitivity analysis.

Parameter	Description	Value	Reasoning
Δ	Lattice spacing	10μm	T and B cell sizes range from 8-10 μm Rodgers et al. (2013), with 10μm assumed as a uniform diameter.
τ	Time step duration	20 minutes	TCR-HaloTag cells have an average diffusion coefficient of $0.082\mu m^2/s$ Chen et al. (2021). We assume that cells move one body length per time step, so that $100\mu m^2/(0.082 * 60) = \tau \approx 20$ mins.
~	Relapse duration	4 weeks	Relapsing episodes are days to weeks in duration Cree et al. (2019).
~	Relapse frequency	Every 100 days from the onset of the previous relapse	Diagnostic criteria suggests that the onset of two distinct relapses are separated by at least 30 days McDonald et al. (2001).
~	Total window simulated	300 days/ 21600 time steps	Allows us to simulate multiple relapses while maintaining a reasonable simulation time.
~	Height of domain	3000μm/ 3mm/ 300 units	Small MS lesions are typically at least 3mm (3000μm) along their major axis Filippi et al. (2019).
~	Width of peripheral blood	30μm / 3 units	Blood vessels range in size from 25,000 μm for an aorta to 8 μm for a capillary Müller et al. (2008). The diameters of venules and arterioles are 20 μm - 30 μm respectively.
~	Width of perivascular space	50 μm / 5 units	We assume an enlarged perivascular space, adopting the width used in Moise and Friedman (2021).

~	Width of perenchyma	1000 μm / 100 units	Given the height of the domain is 3000 μm this width allows us to model over a 3mm^2 myelinated area.
~	Thickness of the blood brain barrier	0μm / 0 units	The BBB is anatomically represented by the endothelial membrane which is 0.3 - 0.5 μm thick Zlokovic (2009). This is negligible in the scale of our model.
ρ_R	Primed T cell renewal rate (relapsing rate)	0.0025	Chosen to give rise to a downstream activated T cell density of 30-40 cells/mm^2 in the parenchyma.
ρ_{NR}	Primed T cell renewal rate (non-relapsing rate)	0.0002	Chosen to give rise to a reduced, non-zero activated T cell presence of 1-2 cells/mm^2 in the parenchyma.
ρ_d	Decay of T cells (primed and reactivated T cells)	0.35/day	The estimated half life of cytotoxic T lymphocytes (CTLs) is 48 hours, to give a decay rate of 0.35/day Jenner et al. (2018); Kim et al. (2011).
~	Perivascular macrophage density	300 cells/mm^2	1-4% of the blood vessel surface was shown to be covered by PVMs Karam et al. (2022). We estimate a PVM density of 300 cells/mm^2 in the PVS by assuming 3% occupation of the 1500 (5×300) lattice sites that comprise the 0.15mm^2 area of PVS.
~	Oligodendrocyte density/ size	400 cells/mm^2 (5×5 lattice site area)	Surface area of an individual oligodendrocyte's myelin membrane depends on its type and can reach 50,000 μm^2 Simons and Nave (2016). Given such broad estimates in the literature, we follow the models of Khonsari and Calvez (2007) and Lombardo et al. (2017) to assume an oligodendrocyte density of 400 cells per mm^2 Lucchinetti et al. (2000), equivalent to 2500 μm^2 of coverage per oligodendrocyte.

β	Maximum chemotaxis strength of activated T cells	0.5	Set so that the total bias in an individual direction is less than 100 percent, imposing a weak trial and error process along the biochemical gradient.
k	Scaling parameter for bias decay	0.025	A small, positive constant.
$\mathcal{S}$	Number of myelin states	5	Estimated. Choice of 5 states means it takes 4*τ = 80 mins for an activated T cell to fully degrade one myelinated site.
$\mathcal{W}$	Time to heal myelin by one state	25τ = 8 hours, 20 mins	Estimated. Chosen so that the total time to repair a myelin site is greater than the time to degrade it.
b_R	Blood-brain barrier permeability	(sensitivity analysis)	Required to be a nonzero probability to allow T cell migration into the CNS.
ω	Remyelination threshold	(sensitivity analysis)	Number of damaged myelin sites before oligodendrocyte stops synthesising/repairing myelin.
λ	Apoptosis threshold	(sensitivity analysis)	Number of damaged myelin sites before oligodendrocyte undergoes apoptosis.

Table A

Model implementation and costs

The model is developed in Matlab. All code is available on Github. The structure of the main algorithm is given in Alg A,B. We provide two versions of the algorithm for the reader's convenience. We have specified seeds for each simulation to ensure that our results are reproducible. The average runtime for one simulation of the model with the parameters shown in Table A and $b_R = 0.1, \omega = 10$, and $\lambda = 14$ is 2 mins 24 sec (averaged across 5 simulations on MacBook Air M2). While several simulations of the model are able to be run in a timely manner, high volumes of simulations were supported by the high-performance computing facility of the Queensland University of Technology eResearch Office.

Algorithm A ABM algorithm

```
Require: Setup initial conditions and domain/subdomains as per paper description.
for t = 1 to T in time-step τ do
    if damage to myelin agents then
        check corresponding oligodendrocyte agent states
        if oligodendrocyte agents are myelinating then
            repair myelin agents if enough time steps passed since damage
        else
            leave myelin agents damaged
        end if
    end if
    if reactivated T cells agents exist then
        if myelin agents all intact then
            move reactivated T cells agents in unbiased random walk
        else if damage to myelin agent/s then
            bias each reactivated T cell agent towards the nearest myelin agent
        end if
    end if
    move primed T cells agents in unbiased random walk
    if primed T cell agent attempts to cross blood-brain barrier to enter perivascular space then
        if primed T cell agent satisfies boundary condition then
            move primed T cell agent to target site in perivascular space
        else
            primed T cell agent remains in the peripheral blood
        end if
    end if
    move perivascular macrophage agents in unbiased random walk
    if primed T cell, reactivated T cell or perivascular macrophage agents have exited boundary then
        enforce boundary condition and update agent positions
    end if
    if primed T cell agent and perivascular macrophage agent occupy same lattice site then
        create new reactivated T cell agent at lattice site
        remove primed T cell agent from simulation
    end if
    if myelin agent/s occupied by reactivated T cell agent then
        record damage to myelin agent
    end if
    if damage to an oligodendrocyte agent's myelin block exceeds user-set level then
        update oligodendrocyte agent's behaviour
        if oligodendrocyte agent undergoes apoptosis then
            update all of its myelin agents to be fully damaged
        end if
    end if
    if in a pre-defined relapse event then
        add primed T cells at relapse rate
    else
        add primed T cells at non-relapse rate
    end if
    if death events of primed T cell agents or reactivated T cell agents then
        remove cells from the simulation
    end if
end for
```

Algorithm B ABM algorithm

Require: Setup initial conditions and domain/subdomains as per paper description.

for t = 1 to T in time-step τ **do**
  if $\exists$ myelin agents j with state M_i where $i \in \{1, 2, ..., \mathcal{S} - 1\}$ **then**
    if $\sigma < \omega$ for corresponding oligodendrocyte and (time since damage mod $\mathcal{W}$) = 0 **then**
      repair myelin agents j from state M_i to state M_{i+1}
    else $\sigma \geq \omega$
      leave myelin agents damaged with state M_i
    end if
  end if
  if reactivated T cells agents exist **then**
    if $\forall$ myelin agents j with state $M_{\mathcal{S}}$ **then**
      move reactivated T cells agents in unbiased random walk
    else if $\forall$ myelin agents j with state M_i where $i \in \{1, 2, ..., \mathcal{S} - 1\}$ **then**
      moved reactivated T cell agent in biased walk towards myelin agent j with bias strength s_{bias}
    end if
  end if
  move primed T cells agents in unbiased random walk
  if primed T cell agent attempts to cross blood-brain barrier to enter perivascular space **then**
    if $b_R > r \sim U(0, 1)$ **then**
      move primed T cell agent to target site in perivascular space
    else $b_R \leq r \sim U(0, 1)$
      primed T cell agent remains in the peripheral blood
    end if
  end if
  move perivascular macrophage agents in unbiased random walk
  if primed T cell, reactivated T cell or perivascular macrophage agents have exited boundary **then**
    enforce boundary condition and update agent positions
  end if
  if primed T cell agent and perivascular macrophage agent occupy same lattice site **then**
    create new reactivated T cell agent at lattice site and remove primed T cell agent
  end if
  if myelin agent/s occupied by reactivated T cell agent **then**
    record damage to myelin agent
  end if
  if $\sigma \geq \lambda$ **then**
    update oligodendrocyte agent to have undergone apoptosis
    if oligodendrocyte agent undergoes apoptosis **then**
      Enforce that the oligodendrocyte's myelin agents j have state M_1
    end if
  end if
  if in a predefined relapse event **then**
    if $\rho_R > r \sim U(0, 1)$ **then**
      add primed T cells
    end if
  else not in a relapse event
    if $\rho_{NR} > r \sim U(0, 1)$ **then**
      add primed T cells
    end if
  end if
  if $\forall$ T cell agents and activated T cell agents satisfying $\rho_d > r \sim U(0, 1)$ **then**
    remove cells from the simulation
  end if
end for

Simulation averaging

Each simulation of the model is assigned a unique random seed. We are interested in averaged behaviour in addition to individual trajectories. Here, we show the averaged results as the number of simulations is increased. We show reactivated T cell counts and myelin counts for Days 120, 170, 220 and 270. The reactivated T cell count only begins to settle from around 80 simulations and onward (see Figure A). The averaged results for intact myelin are comparatively less noisy (Figure B). Consistent results are obtained by averaging 20+ simulations. In the interest of conserving computational effort, we choose to simulate across 40 simulations, allowing some settling of the reactivated T cell population and consistency in the myelin results.

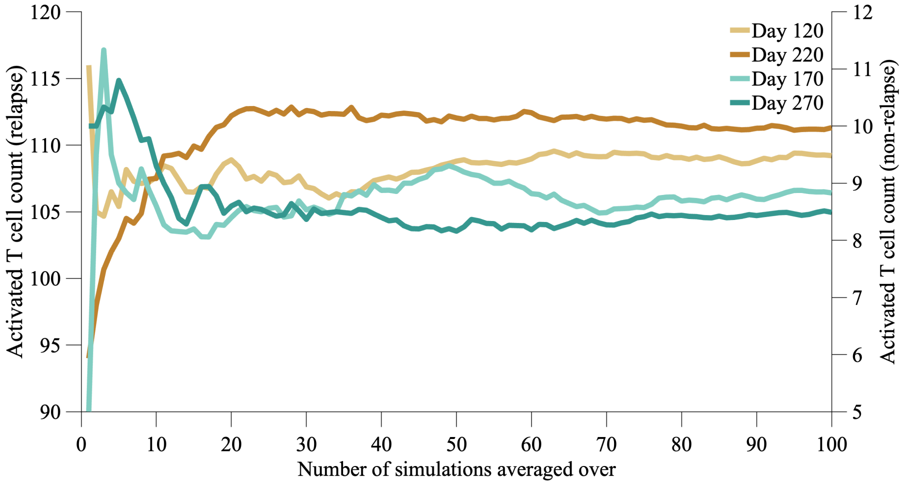

Figure A: Average reactivated T cell population at Days 120, 170, 220 and 270. As we increase the number of simulations averaged across, we achieve more consistent averaged behaviour in the reactivated T cell data. Note that two axes are shown: relapsing times (Days 120, 220) on the left axis, nonrelapsing times (Days 170,270) on the right right axis

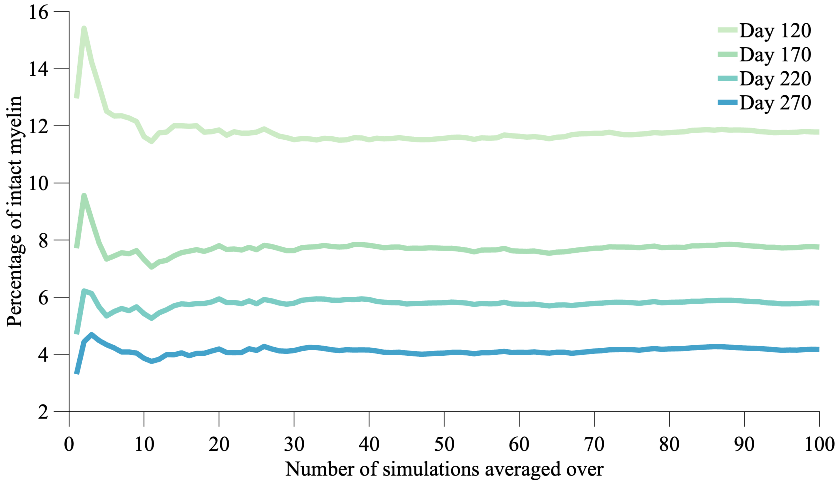

Figure B: Average percentage of intact myelin at Days 120, 170, 220 and 270. As we increase the number of simulations averaged across, we achieve more consistent averaged behaviour in the myelin data.

Biased movement cases

We define all expressions for the biased movement of a reactivated T cell agent towards a myelinated lattice site as follows. Please note that the directions are labelled according to where the myelinated lattice site is relative to the reactivated T cell that is being biased.

North:

$$\begin{aligned}
\Pr\left((x_{\mathcal{T}}, y_{\mathcal{T}}) \to (x_{\mathcal{T}}, y_{\mathcal{T}}+1)\right) &= 0.25 + s_{\text{bias}}, \\
\Pr\left((x_{\mathcal{T}}, y_{\mathcal{T}}) \to (x_{\mathcal{T}}+1, y_{\mathcal{T}})\right) &= 0.25 - \frac{s_{\text{bias}}}{3}, \\
\Pr\left((x_{\mathcal{T}}, y_{\mathcal{T}}) \to (x_{\mathcal{T}}, y_{\mathcal{T}}-1)\right) &= 0.25 - \frac{s_{\text{bias}}}{3}, \\
\Pr\left((x_{\mathcal{T}}, y_{\mathcal{T}}) \to (x_{\mathcal{T}}-1, y_{\mathcal{T}})\right) &= 0.25 - \frac{s_{\text{bias}}}{3}.
\end{aligned}$$

East:

$$\begin{aligned}
\Pr\left((x_{\mathcal{T}}, y_{\mathcal{T}}) \to (x_{\mathcal{T}}, y_{\mathcal{T}}+1)\right) &= 0.25 - \frac{s_{\text{bias}}}{3}, \\
\Pr\left((x_{\mathcal{T}}, y_{\mathcal{T}}) \to (x_{\mathcal{T}}+1, y_{\mathcal{T}})\right) &= 0.25 + s_{\text{bias}}, \\
\Pr\left((x_{\mathcal{T}}, y_{\mathcal{T}}) \to (x_{\mathcal{T}}, y_{\mathcal{T}}-1)\right) &= 0.25 - \frac{s_{\text{bias}}}{3}, \\
\Pr\left((x_{\mathcal{T}}, y_{\mathcal{T}}) \to (x_{\mathcal{T}}-1, y_{\mathcal{T}})\right) &= 0.25 - \frac{s_{\text{bias}}}{3}.
\end{aligned}$$

South:

$$\begin{aligned}
\Pr\left((x_{\mathcal{T}}, y_{\mathcal{T}}) \to (x_{\mathcal{T}}, y_{\mathcal{T}}+1)\right) &= 0.25 - \frac{s_{\text{bias}}}{3}, \\
\Pr\left((x_{\mathcal{T}}, y_{\mathcal{T}}) \to (x_{\mathcal{T}}+1, y_{\mathcal{T}})\right) &= 0.25 - \frac{s_{\text{bias}}}{3}, \\
\Pr\left((x_{\mathcal{T}}, y_{\mathcal{T}}) \to (x_{\mathcal{T}}, y_{\mathcal{T}}-1)\right) &= 0.25 + s_{\text{bias}}, \\
\Pr\left((x_{\mathcal{T}}, y_{\mathcal{T}}) \to (x_{\mathcal{T}}-1, y_{\mathcal{T}})\right) &= 0.25 - \frac{s_{\text{bias}}}{3}.
\end{aligned}$$

West:

$$\begin{aligned}
\Pr\left((x_{\mathcal{T}}, y_{\mathcal{T}}) \to (x_{\mathcal{T}}, y_{\mathcal{T}}+1)\right) &= 0.25 - \frac{s_{\text{bias}}}{3}, \\
\Pr\left((x_{\mathcal{T}}, y_{\mathcal{T}}) \to (x_{\mathcal{T}}+1, y_{\mathcal{T}})\right) &= 0.25 - \frac{s_{\text{bias}}}{3}, \\
\Pr\left((x_{\mathcal{T}}, y_{\mathcal{T}}) \to (x_{\mathcal{T}}, y_{\mathcal{T}}-1)\right) &= 0.25 - \frac{s_{\text{bias}}}{3}, \\
\Pr\left((x_{\mathcal{T}}, y_{\mathcal{T}}) \to (x_{\mathcal{T}}-1, y_{\mathcal{T}})\right) &= 0.25 + s_{\text{bias}}.
\end{aligned}$$

North-East:

$$\begin{aligned}
\Pr\left((x_{\mathcal{T}},y_{\mathcal{T}})\to(x_{\mathcal{T}},y_{\mathcal{T}}+1)\right)&=0.25+s_{\text{bias}}\left(\frac{|y_{\mathcal{T}}-y_j|}{|x_{\mathcal{T}}-x_j|+|y_{\mathcal{T}}-y_j|}\right),\\
\Pr\left((x_{\mathcal{T}},y_{\mathcal{T}})\to(x_{\mathcal{T}}+1,y_{\mathcal{T}})\right)&=0.25+s_{\text{bias}}\left(\frac{|x_{\mathcal{T}}-x_j|}{|x_{\mathcal{T}}-x_j|+|y_{\mathcal{T}}-y_j|}\right),,\\
\Pr\left((x_{\mathcal{T}},y_{\mathcal{T}})\to(x_{\mathcal{T}},y_{\mathcal{T}}-1)\right)&=0.25-\frac{s_{\text{bias}}}{2},\\
\Pr\left((x_{\mathcal{T}},y_{\mathcal{T}})\to(x_{\mathcal{T}}-1,y_{\mathcal{T}})\right)&=0.25-\frac{s_{\text{bias}}}{2}.
\end{aligned}$$

North-West:

$$\begin{aligned}
\Pr\left((x_{\mathcal{T}},y_{\mathcal{T}})\to(x_{\mathcal{T}},y_{\mathcal{T}}+1)\right)&=0.25+s_{\text{bias}}\left(\frac{|y_{\mathcal{T}}-y_j|}{|x_{\mathcal{T}}-x_j|+|y_{\mathcal{T}}-y_j|}\right),\\
\Pr\left((x_{\mathcal{T}},y_{\mathcal{T}})\to(x_{\mathcal{T}}+1,y_{\mathcal{T}})\right)&=0.25-\frac{s_{\text{bias}}}{2},\\
\Pr\left((x_{\mathcal{T}},y_{\mathcal{T}})\to(x_{\mathcal{T}},y_{\mathcal{T}}-1)\right)&=0.25-\frac{s_{\text{bias}}}{2},\\
\Pr\left((x_{\mathcal{T}},y_{\mathcal{T}})\to(x_{\mathcal{T}}-1,y_{\mathcal{T}})\right)&=0.25+s_{\text{bias}}\left(\frac{|x_{\mathcal{T}}-x_j|}{|x_{\mathcal{T}}-x_j|+|y_{\mathcal{T}}-y_j|}\right).
\end{aligned}$$

South-East:

$$\begin{aligned}
\Pr\left((x_{\mathcal{T}},y_{\mathcal{T}})\to(x_{\mathcal{T}},y_{\mathcal{T}}+1)\right)&=0.25-\frac{s_{\text{bias}}}{2},\\
\Pr\left((x_{\mathcal{T}},y_{\mathcal{T}})\to(x_{\mathcal{T}}+1,y_{\mathcal{T}})\right)&=0.25+s_{\text{bias}}\left(\frac{|x_{\mathcal{T}}-x_j|}{|x_{\mathcal{T}}-x_j|+|y_{\mathcal{T}}-y_j|}\right),\\
\Pr\left((x_{\mathcal{T}},y_{\mathcal{T}})\to(x_{\mathcal{T}},y_{\mathcal{T}}-1)\right)&=0.25+s_{\text{bias}}\left(\frac{|y_{\mathcal{T}}-y_j|}{|x_{\mathcal{T}}-x_j|+|y_{\mathcal{T}}-y_j|}\right),\\
\Pr\left((x_{\mathcal{T}},y_{\mathcal{T}})\to(x_{\mathcal{T}}-1,y_{\mathcal{T}})\right)&=0.25-\frac{s_{\text{bias}}}{2}.
\end{aligned}$$

South-West:

$$\begin{aligned}
\Pr\left((x_{\mathcal{T}},y_{\mathcal{T}})\to(x_{\mathcal{T}},y_{\mathcal{T}}+1)\right)&=0.25-\frac{s_{\text{bias}}}{2},\\
\Pr\left((x_{\mathcal{T}},y_{\mathcal{T}})\to(x_{\mathcal{T}}+1,y_{\mathcal{T}})\right)&=0.25-\frac{s_{\text{bias}}}{2},\\
\Pr\left((x_{\mathcal{T}},y_{\mathcal{T}})\to(x_{\mathcal{T}},y_{\mathcal{T}}-1)\right)&=0.25+s_{\text{bias}}\left(\frac{|y_{\mathcal{T}}-y_j|}{|x_{\mathcal{T}}-x_j|+|y_{\mathcal{T}}-y_j|}\right),\\
\Pr\left((x_{\mathcal{T}},y_{\mathcal{T}})\to(x_{\mathcal{T}}-1,y_{\mathcal{T}})\right)&=0.25+s_{\text{bias}}\left(\frac{|x_{\mathcal{T}}-x_j|}{|x_{\mathcal{T}}-x_j|+|y_{\mathcal{T}}-y_j|}\right).
\end{aligned}$$

Stochastic spatial realisations

Here we provide further spatial snapshots of the model. We show four different stochastic realisations of the model at Day 10 and Day 20 of the simulation. We simulated with the following parameters: $b_R = 0.1, \omega = 10$, and $\lambda = 14$. All other parameter choices follow those specified in S1 Full parameter table.

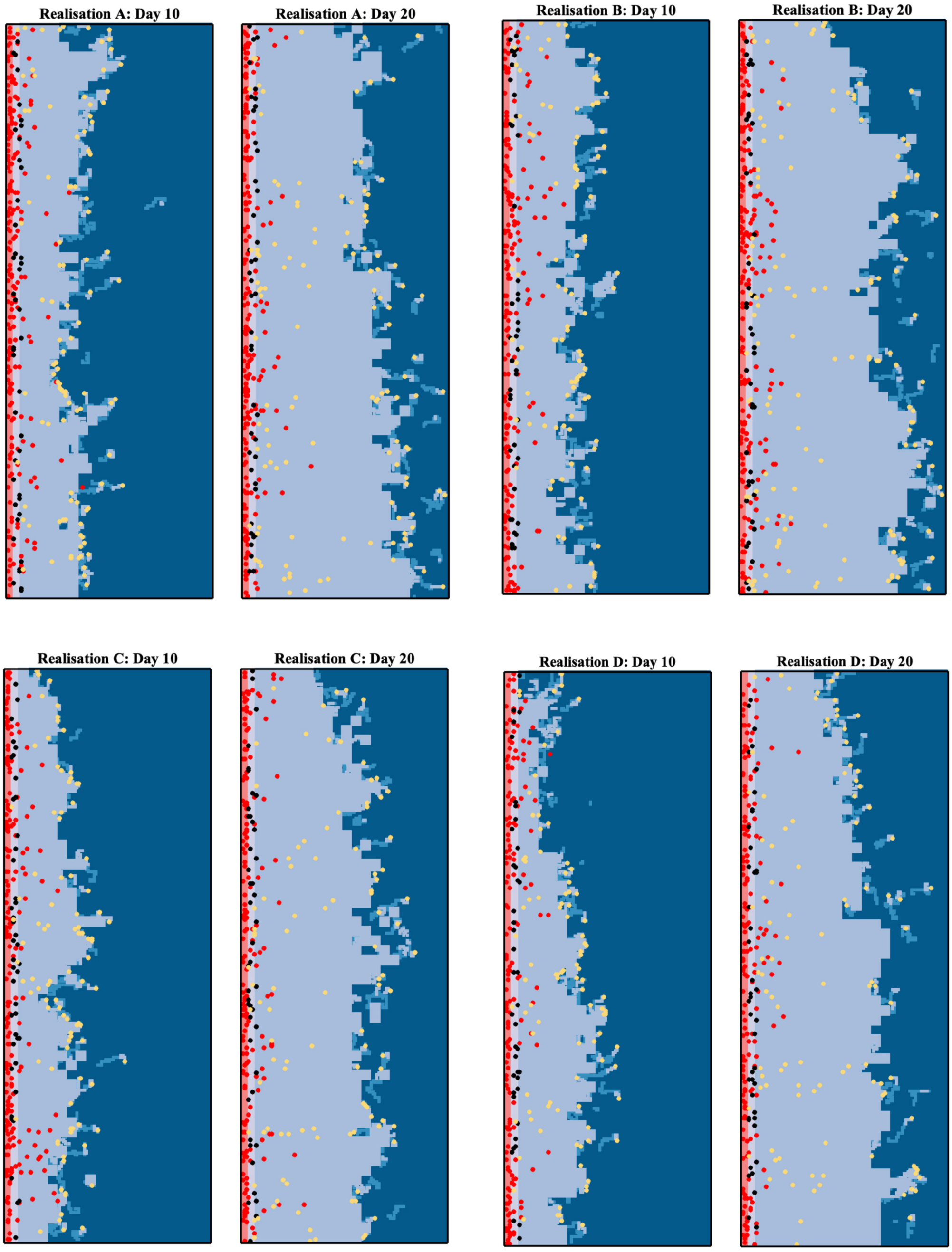

Figure C: Spatial insights from four individual realisations of the model (labelled A,B,C,D respectively). Shown at Day 10 and Day 20, the model produces rightward-progressing lesions (light blue). The progression and heterogeneity of the lesion boundary are driven by the reactivated T cell population (yellow). These reactivated T cells create paths of intermediate damage (blue) in the densely myelinated region (dark blue).

Relapse schedules

Throughout the main text we simulate three, 28 day relapses over a 300 day window. Here, we outline three additional relapse schedules and simulate the combined treatment. Please note that the original relapse schedule is listed as relapse schedule 1.

- Relapse Schedule 1: 3 relapses, 28 days each, 84 total relapse days
- Relapse Schedule 2: 3 relapses, 60 days each, 180 total relapse days
- Relapse Schedule 3: 6 relapses, 14 days each, 84 total relapse days
- Relapse Schedule 4: 6 relapses, 28 days each, 168 total relapse days

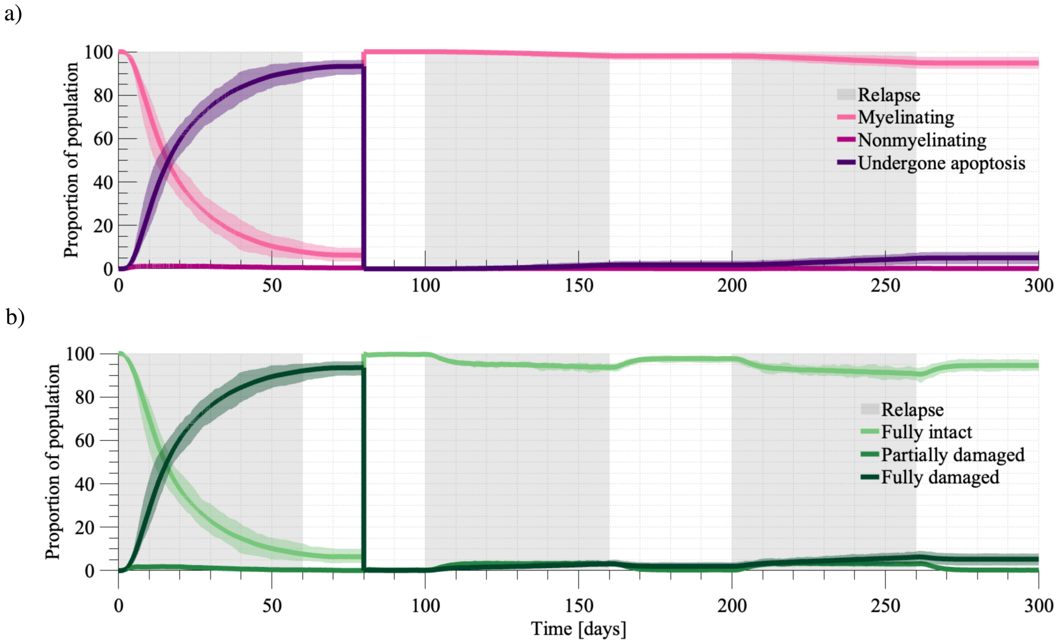

Figure D: Combined treatment results for relapse schedule 2. There are three, 60 day relapses. The combined treatment approach achieves suppressed immune activity through a DMT, and the promotion of remyelination by oligodendrocyte replenishment and increases in their innate resilience. **a** Breakdown of the oligodendrocyte agent population into myelinating, nonmyelinating and having undergone apoptosis. **b** Breakdown of the myelin agent population by its level of damage (fully intact, partially damaged, fully damaged). Initially we show an untreated case where $b_R = 0.1, \omega = 10$, and $\lambda = 14$. Rapid myelin loss motivates the treatment intervention at Day 80, where in addition to restoring the myelin and oligodendrocyte populations we simulate under $b_R = 0.025, \omega = 21$, and $\lambda = 24$.

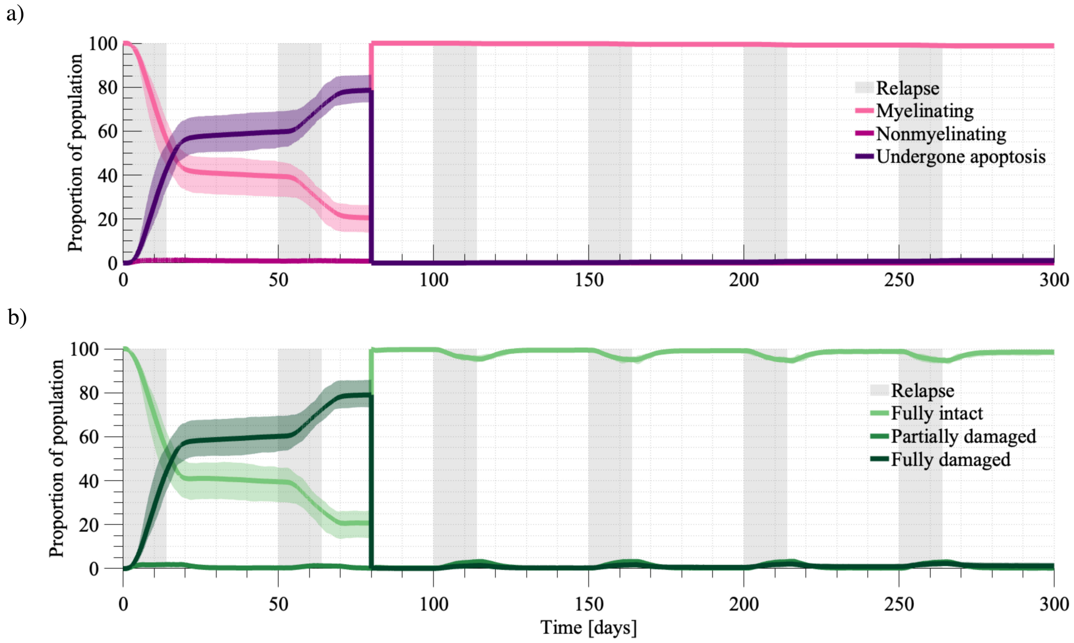

Figure E: Combined treatment results for relapse schedule 3. There are six, 14 day relapses. The combined treatment approach achieves suppressed immune activity through a DMT, and the promotion of remyelination by oligodendrocyte replenishment and increases in their innate resilience. **a** Breakdown of the oligodendrocyte agent population into myelinating, nonmyelinating and having undergone apoptosis. **b** Breakdown of the myelin agent population by its level of damage (fully intact, partially damaged, fully damaged). Initially we show an untreated case where $b_R = 0.1, \omega = 10$, and $\lambda = 14$. Rapid myelin loss motivates the treatment intervention at Day 80, where in addition to restoring the myelin and oligodendrocyte populations we simulate under $b_R = 0.025, \omega = 21$, and $\lambda = 24$.

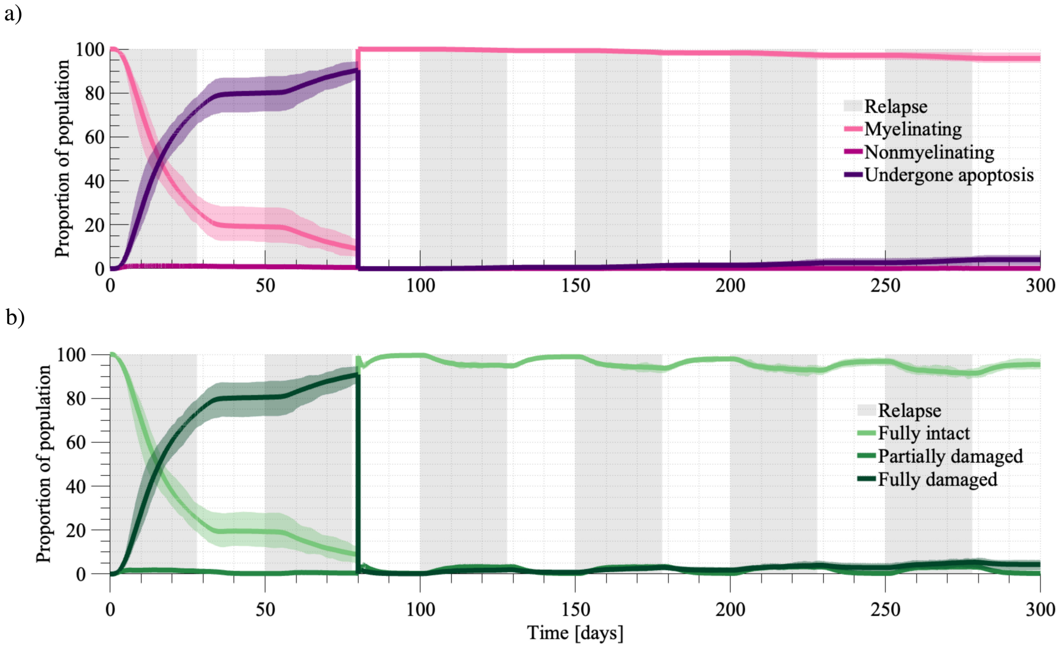

Figure F: Combined treatment results for relapse schedule 4. There are six, 28 day relapses. The combined treatment approach achieves suppressed immune activity through a DMT, and the promotion of remyelination by oligodendrocyte replenishment and increases in their innate resilience. **a** Breakdown of the oligodendrocyte agent population into myelinating, nonmyelinating and having undergone apoptosis. **b** Breakdown of the myelin agent population by its level of damage (fully intact, partially damaged, fully damaged). Initially we show an untreated case where $b_R = 0.1, \omega = 10$, and $\lambda = 14$. Rapid myelin loss motivates the treatment intervention at Day 80, where in addition to restoring the myelin and oligodendrocyte populations we simulate under $b_R = 0.025, \omega = 21$, and $\lambda = 24$.

1 References

Chen, K. Y., Jenkins, E., Körbel, M., Ponjavic, A., Lippert, A. H., Santos, A. M., Ashman, N., O'Brien-Ball, C., McBride, J., Klenerman, D., and Davis, S. J. (2021). Trapping or slowing the diffusion of t cell receptors at close contacts initiates t cell signaling. *Proceedings of the National Academy of Sciences*, 118(39):e2024250118.

Cree, B. A., Hollenbach, J. A., Bove, R., Kirkish, G., Sacco, S., Caverzasi, E., Bischof, A., Gundel, T., Zhu, A. H., et al. (2019). Silent progression in disease activity–free relapsing multiple sclerosis. *Annals of neurology*, 85(5):653–666.

Filippi, M., Preziosa, P., Banwell, B. L., Barkhof, F., Ciccarelli, O., De Stefano, N., Geurts, J. J., Paul, F., Reich, D. S., Toosy, A. T., et al. (2019). Assessment of lesions on magnetic resonance imaging in multiple sclerosis: practical guidelines. *Brain*, 142(7):1858–1875.

Jenner, A. L., Yun, C.-O., Yoon, A., Coster, A. C., and Kim, P. S. (2018). Modelling combined virotherapy and immunotherapy: strengthening the antitumour immune response mediated by il-12 and gm-csf expression. *Letters in Biomathematics*, 5(sup1):S99–S116.

Karam, M., Janbon, H., Malkinson, G., and Brunet, I. (2022). Heterogeneity and developmental dynamics of lyve-1 perivascular macrophages distribution in the mouse brain. *Journal of Cerebral Blood Flow & Metabolism*, 42(10):1797–1812.

Khonsari, R. H. and Calvez, V. (2007). The origins of concentric demyelination: self-organization in the human brain. *PLoS One*, 2(1):e150.

Kim, P. S., Lee, P. P., and Levy, D. (2011). A theory of immunodominance and adaptive regulation. *Bulletin of mathematical biology*, 73(7):1645–1665.

Lombardo, M., Barresi, R., Bilotta, E., Gargano, F., Pantano, P., and Sammartino, M. (2017). Demyelination patterns in a mathematical model of multiple sclerosis. *Journal of mathematical biology*, 75(2):373–417.

Lucchinetti, C., Brück, W., Parisi, J., Scheithauer, B., Rodriguez, M., and Lassmann, H. (2000). Heterogeneity of multiple sclerosis lesions: implications for the pathogenesis of demyelination. *Annals of Neurology: Official Journal of the American Neurological Association and the Child Neurology Society*, 47(6):707–717.

McDonald, W. I., Compston, A., Edan, G., Goodkin, D., Hartung, H.-P., Lublin, F. D., McFarland, H. F., Paty, D. W., Polman, C. H., Reingold, S. C., et al. (2001). Recommended diagnostic criteria for multiple sclerosis: guidelines from the international panel on the diagnosis of multiple sclerosis. *Annals of Neurology: Official Journal of the American Neurological Association and the Child Neurology Society*, 50(1):121–127.

Moise, N. and Friedman, A. (2021). A mathematical model of the multiple sclerosis plaque. *Journal of Theoretical Biology*, 512:110532.

Müller, B., Lang, S., Dominietto, M., Rudin, M., Schulz, G., Deyhle, H., Germann, M., Pfeiffer, F., David, C., and Weitkamp, T. (2008). High-resolution tomographic imaging of microvessels. In *Developments in X-ray tomography VI*, volume 7078, pages 89–98. SPIE.

Rodgers, J. M., Robinson, A. P., and Miller, S. D. (2013). Strategies for protecting oligodendrocytes and enhancing remyelination in multiple sclerosis. *Discovery medicine*, 16(86):53–63.

Simons, M. and Nave, K.-A. (2016). Oligodendrocytes: myelination and axonal support. *Cold Spring Harbor perspectives in biology*, 8(1):a020479.

Zlokovic, B. (2009). *Blood–Brain Barrier and Neurovascular Mechanisms of Neurodegeneration and Injury*, pages 265–271.
